# Laboratory Comparison of Rapid Antigen Diagnostic Tests for Lymphatic Filariasis: STANDARD Q Filariasis Antigen Test (QFAT) Versus Bioline Filariasis Test Strip (FTS)

**DOI:** 10.3390/tropicalmed10010023

**Published:** 2025-01-14

**Authors:** Patricia M. Graves, Jessica L. Scott, Alvaro Berg Soto, Antin Y. N. Widi, Maxine Whittaker, Colleen L. Lau, Kimberly Y. Won

**Affiliations:** 1 WHO Collaborating Centre for Vector-Borne and Neglected Tropical Diseases, College of Public Health, Medical and Veterinary Sciences and Australian Institute of Tropical Health and Medicine, James Cook University, Cairns, QLD 4878, Australia; jessica.scott2@my.jcu.edu.au (J.L.S.); alvaro_berg@hotmail.com (A.B.S.);; 2 Andes Analytics, Santiago 7560408, Chile; 3 Faculty of Medicine and Veterinary Medicine, Nusa Cendana University, Kupang 85228, East Nusa Tenggara, Indonesia; 4 College of Public Health, Medical and Veterinary Sciences, James Cook University, Townsville, QLD 4811, Australia; 5 School of Public Health, Faculty of Medicine, The University of Queensland, Brisbane, QLD 4006, Australia; 6 Centre for Clinical Research, Faculty of Medicine, The University of Queensland, Brisbane, QLD 4006, Australia; 7 Division of Parasitic Diseases and Malaria, Centers for Disease Control and Prevention, Atlanta, GA 30333, USA

**Keywords:** Filariasis, diagnostics, antigen, microfilaria, surveillance, *Wuchereria bancrofti*, elimination

## Abstract

Accurate rapid diagnostic tests (RDTs) are needed to diagnose lymphatic filariasis (LF) in global elimination programmes. We evaluated the performance of the new STANDARD Q Filariasis Antigen Test (QFAT) against the Bioline Filariasis Test Strip (FTS) for detecting *W. bancrofti* antigen (Ag) in laboratory conditions, using serum (n = 195) and plasma (n = 189) from LF-endemic areas (Samoa, American Samoa and Myanmar) and Australian negative controls (n = 46). The prior Ag status of endemic samples (54.9% Ag-positive) was determined by rapid test (ICT or FTS) or Og4C3 ELISA. The proportion of samples testing positive at 10 min was similar for QFAT (44.8%) and FTS (41.3%). Concordance between tests was 93.5% (kappa 0.87, n = 417) at 10 min, and it increased to 98.8% (kappa 0.98) at 24 h. The sensitivities of QFAT and FTS at 10 min compared to the prior results were 92% (95% CI 88.0–96.0) and 86% (95% CI 80.0–90.0), respectively, and they increased to 97% and 99% at 24 h. Specificity was 98% for QFAT and 99% for FTS at 10 min. Both tests showed evidence of cross-reaction with *Dirofilaria repens* and *Onchocerca lupi* but not with *Acanthochilonema reconditum* or *Cercopithifilaria bainae.* Under laboratory conditions, QFAT is a suitable alternative RDT to FTS.

## 1. Introduction

Lymphatic filariasis (LF) is a mosquito-transmitted neglected tropical disease (NTD) caused by infection with filarial parasites (*Wuchereria bancrofti*, *Brugia malayi*, or *B. timori*). Over time, infection can damage lymphatic vessels, causing hydrocele and lymphoedema. People who live with these chronic and disabling manifestations of LF can experience reduced economic productivity and social stigma. The Global Programme to Eliminate Lymphatic Filariasis (GPELF), established by the World Health Organization (WHO), aims to eliminate LF as a public health problem with a two-armed approach: interrupting transmission through mass drug administration (MDA) of anti-filarial medicines and alleviating suffering among patients through morbidity management and disability prevention [1]. Currently, 39 of 72 LF-endemic countries still need MDA with the majority implementing it with support from GPELF [1]. There has been a 74% reduction in global LF infections from 1997 to 2018 [2], but many countries are still conducting MDA or surveillance to monitor progress toward elimination.

National programs require diagnostic tests to establish baseline endemicity, monitor the impact of MDA campaigns, inform decisions to stop MDA in transmission assessment surveys (TAS), and detect potential recrudescent transmission post-cessation of MDA [3,4]. Therefore, access to rapid, accurate (sensitive and specific), and user-friendly diagnostic tools are important criteria of the target product profiles of the Diagnostic Technical Advisory Group for NTDs (DTAG-NTD), which should be considered when recommending tests for implementation into surveillance programmes [5].

Historically, the gold standard test for diagnosing LF was identifying microfilariae (Mf) on stained blood slides, but this requires time and skill, and (outside the Pacific areas of subperiodic diurnal transmission) blood samples for slides must be collected at night. In the 1980s, two enzyme-linked immunosorbent assays (ELISAs) were developed to detect *W. bancrofti* using monoclonal antibodies to detect the same circulating filarial antigen (Ag) from adult worms [6,7,8]. Both tests produce quantitative readouts and can be performed using samples derived from either dried blood spots, anticoagulated whole blood, serum or plasma with attention to dilution factors [9]. However, these tests require a suitably equipped laboratory and cannot be deployed readily during LF field surveys.

One of the Ag ELISA tests, Og4C3, is commercially available (Cellabs, Sydney, Australia). The second ELISA test mentioned above [8] was subsequently converted to a rapid diagnostic test (RDT) (immunochomatographic test (ICT)) (initially Binax Now) [10], which was widely utilised at the start of GPELF national surveys. Subsequently, to reduce the cost and amount of blood needed for ICT, and improve the shelf life and storage requirements, the Alere (now Abbott) Filariasis Test Strip (FTS) was developed using the same critical reagents but in a new test format [11]. Despite some discrepancies in concordance between the ICT and FTS tests in field evaluations (reviewed in [12]) with FTS showing increased sensitivity, the WHO was satisfied with the diagnostic characteristics of FTS compared with ICT [13]. Thus, guidance on implementing TAS and critical cut-off numbers for passing were not changed, and the FTS test was approved for use in programme monitoring.

Since 2015, the FTS has been the primary Ag rapid diagnostic test recommended for use in areas where *W. bancrofti* is the causative agent of LF. However, several limitations related to its use have been identified. Previous studies have reported test cross-reactivity with *Loa loa* infections [14,15], although this worm is not found in the Asia–Pacific region. The FTS requires a relatively large volume of blood (75 µL), which is usually collected via finger prick. The time needed to collect this volume at the point of care level could increase the risk of clotting, which can impede blood flow through the test strip and render the results invalid, necessitating repeat sampling and testing. In LF surveys, it is common practice to collect finger prick blood into tubes with anticoagulants for later testing, and the choice of anticoagulant is important, since FTS is thought to be less accurate when used with EDTA than heparinised blood samples. Additionally, issues with capillary action that allow the blood to flow through the test often require modifications to the manufacturer’s recommended procedure, such as delaying the start of the 10-min reading period, which may affect result accuracy. The test format of FTS also presents logistical challenges. The test strip, which lacks protective housing, must be secured with tape to a plastic tray during testing. This cumbersome setup can delay sample processing and increase the potential for user error. Therefore, an improved and more user-friendly alternative rapid antigen test is urgently needed. Moreover, the COVID-19 pandemic caused RDT manufacturers to redirect production towards the SARS-CoV-2 Ag tests, resulting in global supply problems for FTS [16]. As the only reliable test available for LF surveys at the time, this shortage further hindered surveillance programmes in addition to challenges caused by the pandemic. The availability of an alternative LF Ag test could therefore also alleviate supply issues in the future.

The STANDARD Q Filariasis Antigen Test (QFAT) (SD Biosensor, Suwon, Republic of Korea), a new RDT for filarial Ag, has been proposed as an alternative for use in LF monitoring and surveillance programmes. The test uses the same methodology as FTS (capture of LF antigen in blood using a monoclonal antibody immobilized on a test strip, visualized by conjugated secondary antibodies). It has already been trialled in Samoa, which is an endemic country for *W. bancrofti* [17] as well as India [18]. The field laboratory studies found that QFAT had a similar performance to FTS with high levels of agreement between both tests in detecting LF Ag in finger prick whole blood samples. In the Samoa field laboratory study, users preferred QFAT over FTS primarily due to its reduced sample volume requirements, ease of use and improved readability [17]. However, further studies evaluating QFAT’s performance in other LF-endemic countries are required to support its recommendation as an alternative diagnostic test to use in LF surveillance programmes. A key limitation of the previous field laboratory studies was the inability to assess test sensitivity and specificity against a reference standard, as Ag status was not confirmed with other prior assays at the time of the study. Moreover, the scope of the studies did not include samples from individuals without LF exposure or those with known infection with other helminths. This current study addresses these gaps by utilising archived samples with previously characterised Ag status, samples from non-endemic areas, and those with other helminth infections.

Although LF rapid Ag tests are designed primarily to give a binary (positive/negative) result, they could also provide a semi-quantitative readout by comparing the intensity of the test line to the control line. Previously, it was demonstrated that the FTS test line score was correlated to Ag levels by Og4C3 ELISA, and dark score lines were associated with Mf-slide positivity [19], suggesting that RDTs could be used to indirectly assess the impact of control measures and progress to elimination for LF and similar NTDs like onchocerciasis [20]. Semi-quantitative intensity scoring is frequently used in LF surveys, but its usefulness in cross-sectional studies or with QFAT tests is not yet clear.

During national LF surveys, it is common practice to process multiple samples simultaneously to test for LF Ag. This high throughput means that often, not all tests can be read reliably within the 10-min reading frame. Extending the reading time without compromising test specificity would improve field laboratory workload during high demand, especially for duplicate readers, and allow later checking by supervisors, which could enhance the accuracy of Ag prevalence estimates.

The primary aim of this study was to evaluate the diagnostic performance of QFAT compared to the currently recommended FTS through a head-to-head comparison of samples collected from the Asia–Pacific region with known prior Ag results. Findings from this study will provide further support for the WHO’s decision that the new QFAT can serve as a suitable alternative to the FTS in GPELF activities. Secondary objectives were to investigate the concordance of results between independent test readers, analyse the performance of the tests with different sample types (serum, heparin plasma, EDTA plasma), and investigate cross-reactivity with other helminths. In addition, we studied whether test line intensity was similar between tests and whether there were changes in test results (concordance, sensitivity and specificity) over time.

## 2. Materials and Methods

### 2.1. QFAT and FTS Kits Available for Study

The WHO provided 510 FTS tests (17 boxes of 30 tests) and SD Biosensor, Suwon, Republic of Korea provided 500 QFAT tests (20 boxes of 25 tests) for independent evaluation of test performance. Tests were kept at room temperature (~25 °C) for up to three months before use. The batch numbers were FTS lot number 181193; FTS pouch number 178066; QFAT lot number SIJ35HIAC and QFAT buffer number 5IJ34D1S5. Expiry dates were 28 December 2022 for FTS and 16 January 2024 for QFAT. The study was performed in August 2022. For quality assurance, one of each test type was validated using a positive control (Filariasis Research Reagent Repository, University of Georgia, Athens, GA; https://www.niaid.nih.gov/research/filariasis-research-reagent-resource-center). Additionally, testing with the positive control was repeated with one of each test type at the end of the study. All tests passed quality assurance.

### 2.2. Ethical Approval

Participants gave written consent for their samples to be stored and used for additional studies, except for samples from Myanmar, where waiver of consent for re-use of deidentified samples was applied for and granted. Samples were collected under the following ethical approvals:

▪ American Samoa 2014: Institutional Review Board of American Samoa, James Cook University Human Research Ethics Committee and The University of Queensland (approval number 2014000409).
▪ American Samoa 2016: Australian National University (protocol number 2016/482).
▪ Samoa 2019: Samoa Ministry of Health and The Australian National University Human Research Ethics Committee (protocol number 2018/341).
▪ Myanmar 2014: James Cook University Human Research Ethics Committee approval number H5261 approved by the Ministry of Health and Sports, Myanmar. Since consent in the initial study for future sample use was not fully explicit and participants could not be recontacted, waiver of consent was granted by James Cook University Human Research Ethics Committee (approval number H8341) in consultation with Myanmar collaborators.

### 2.3. Selection Strategy of Serum and Plasma Samples to Use for FTS and QFAT Evaluation

A total of 456 serum and plasma samples were assembled from archived collections from previous surveys where LF is endemic (n = 384) and where LF is non-endemic (n = 72). Samples were selected to have approximately equal proportions of Ag-positive and Ag-negative samples for optimal estimation of test accuracy.

Archived samples from the LF-endemic countries were those previously tested and classified as positive for LF Ag by ICT, FTS or Og4C3 ELISA. Samples originated from studies previously conducted in American Samoa (n = 257), Samoa (n = 35) and Myanmar (n = 92) [21,22,23,24,25]. Results from prior testing were combined into a ‘composite reference standard’ whereby samples were classified as Ag-positive or Ag-negative by any test (ICT, FTS or Og4C3 ELISA) used in the previous studies. The final selection of samples, based on prior testing, resulted in 54.9% Ag-positives and 45.1% Ag-negatives among the samples from endemic areas (Supplementary Table S1). Plasma/serum samples originating from non-LF endemic locations, which also included samples positive for other helminth infections, served as known LF Ag-negative samples or were used to assess potential cross-reactivity. LF has not been endemic in Australia for almost seven decades, so samples originating from Australia were assumed to be negative for LF Ag. The 72 non-endemic samples were composed of samples from Australia (46 human negative controls and 19 human samples positive for *Strongyloides* spp. antibodies by ELISA against *S. ratti* Ag) and seven dog samples from Italy (two infected with *Cercopithifilaria bainae*, two with *Dirofilaria repens*, one with *Acanthochilonema reconditum*, one with *Onchocerca lupi*, and one negative for helminths).

It should be noted that in the American Samoa 2016 collection, the samples of serum (collected in serum-separating tube (SST) vacutainers) and plasma (collected in heparin tubes) were from the same individuals (n = 62). In the Samoa 2019 collection, 12 individuals had plasma collected in both heparin and EDTA tubes. Details of sample collections are available in previous publications [21,22,23,24,25]. EDTA plasma samples were included to investigate whether QFAT would be a more versatile test than FTS for different anticoagulant types.

The samples used for testing were shipped frozen from the country of origin to Australia and stored at minus 70 °C for three to six years depending on the source. Human control samples had been stored for varying periods from six to over 10 years. Most samples had undergone one previous freeze–thaw cycle.

### 2.4. Preparation of Samples Available for Testing with FTS and QFAT

Of the total 456 samples, two (one heparin and one EDTA plasma) from the LF-endemic group had insufficient volume and were removed from the final selection. Additionally, 21 samples had enough volume for testing by QFAT but not by FTS. These included two samples from the LF-endemic group and 19 from the non-endemic group (18 human *Strongyloides* spp. positive samples and one Australian negative control). Prior to testing, samples were thawed from minus 70 °C, aliquoted and kept at 4 °C until testing the same day.

### 2.5. Preliminary Testing for Sample Volumes

Both FTS and QFAT are intended to be used with whole blood directly from a participant’s finger; however, anticoagulated blood can be used for later testing, and heparin is the recommended anticoagulant for FTS. The QFAT instructions state that serum/plasma samples can be used instead of whole blood, but for FTS, the instructions do not indicate whether serum/plasma could alternatively be used. The recommended volume for whole blood for the FTS test is 75 µL, but current instructions do not specify a volume for serum/plasma. Furthermore, the recommended volumes for QFAT are 20 µL for whole blood and 10 µL for serum/plasma.

Ideally, the tests would be compared with the same sample volume to enable a fair assessment of sensitivity. However, preliminary testing using eight samples of Ag-positive serum/plasma from American Samoa revealed that 75 µL for FTS and 10 µL for QFAT, as per the manufacturers’ recommendations is optimal for achieving valid test results. Briefly, none of the samples tested by FTS flowed when 20 µL of the sample was applied, producing invalid results, while the QFAT produced valid results with 10 and 20 µL. It was not feasible to use 75 µL for both tests due to sample volume availability. Based on these preliminary evaluations, we used 75 µL for FTS and 10 µL for QFAT (manufacturers’ recommendations) for the rest of the samples.

### 2.6. Laboratory Head-to-Head Comparison of FTS and QFAT and Test Interpretations

The aliquoted serum/plasma samples were simultaneously tested using FTS and QFAT. Instead of the pipettes supplied by the kits, calibrated micropipettes were used to transfer the appropriate sample volumes, 75 µL for FTS and 10 µL for QFAT, to the tests’ application pads. The results from each test were read at 10 min, per the manufacturer’s instructions, by two independent blinded readers. The 10-min timer was started when the sample migrated up the strip for FTS, and another was started immediately after adding the buffer for QFAT. Classification of test results as either positive, negative or invalid was made following the criteria outlined by the manufacturers. Tests were deemed invalid if no control line was present in the test interpretation window. Samples with invalid test results at the 10-min reading were repeated if sufficient sample volume remained. Flashlights were used for illumination to assess test lines if needed. To determine whether test results remained stable over time, each blinded reader re-read FTS and QFAT at 1 h and 24 h time points.

Semi-quantification of test line intensity of FTS and QFAT was conducted for all tests classified as Ag-positive by the two blinded readers. This was completed for all time points. The intensity of the test line was semi-quantitatively scored relative to the control line where ‘low’ indicated that the test line intensity was lighter than the control, ‘medium’ where the test line intensity was the same as the control or ‘high’ indicating that the test line was darker than the control.

### 2.7. Data Analysis

Results were recorded on paper, transcribed into Excel and imported into R studio (v. 2023.06.0 Build 421) for analysis. Records with missing or inconsistent information were identified and rechecked against paper records. Selected samples were classified as missing (no serum or plasma in the selected vial) or insufficient if they did not have enough volume for testing. Only samples with valid test results for both FTS and QFAT were included in the following analyses unless stated otherwise.

The proportion of tests with discordant interpretations between the two readers was determined. In this analysis, results were stratified by whether the samples originated from an endemic or non-endemic region and then by sample type (i.e., endemic serum, endemic heparin plasma, endemic EDTA plasma, non-endemic human serum, and non-endemic dog serum). McNemar’s Chi-squared test assessed the difference between FTS and QFAT for discordant results for each sample type; a *p*-value of ≤0.05 denoted statistical significance.

For valid tests, the following rules were used to assign a final result to each sample (positive, negative, or indeterminant) at each time point:

- If both observers agreed on positive or negative, the result was assigned as positive or negative, respectively.
- If the two observers disagreed and a third assessment was made on a repeat test with one observer (either same or different to previous observers) to break the tie, the dominant result was assigned (e.g., a positive result if two out of three assessments were positive).
- If the two observers disagreed and a third assessment (repeat test) was not able to be conducted, the result was classified as indeterminant.

The concordance between FTS and QFAT results (percentage agreement of positive and negative LF Ag status) and the Cohen’s Kappa (K) agreement statistic (calculated using the VCD package for R, version 1.4-11) was determined for each time point (10 min, 1 h and 24 h) and reported with a 95% confidence interval (CI). Classification of K was as follows: poor (K ≤ 0.2), fair (K 0.21–0.40), moderate (K 0.41–0.60), good (K 0.61–0.80) and excellent (K 0.81–1.00). For this analysis, only samples with valid Ag results for both FTS and QFAT were included, and indeterminant results were excluded.

Sensitivity, specificity, and positive and negative predictive values were calculated using epiR package v 2.0.62 for FTS and QFAT, based on the final Ag status determined in this study, compared to the results obtained in previous studies using the composite reference standard as noted in Section 2.3 above. This analysis excludes indeterminant results and was performed for all results obtained at the 10 min, 1 h and 24 h time points to assess whether these parameters changed over time.

The semi-quantitative scoring of the test line intensity relative to the control for both readers over time was reported to determine whether readings remained stable over time. Entries with missing values were removed from the analysis.

### 2.8. FTS and QFAT Useability Under Laboratory Conditions

At the end of the study, the two readers of the tests independently provided written feedback on each test, which was prompted by the following categories: instructions for use, test packaging, test setup, sample volume, control line, test readability, and other comments.

## 3. Results

**Figure 1:**
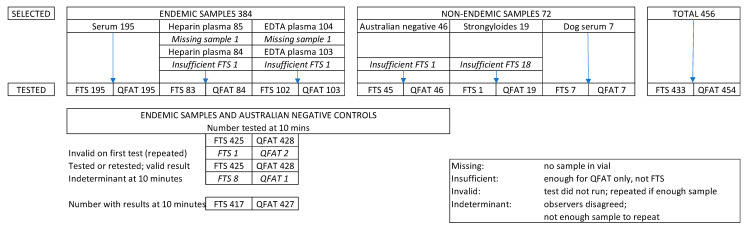
Flowchart of samples selected, tested and indeterminant at the 10-min reading, by sample category and type.

A total of 456 samples were selected for testing, of which 384 were human samples from endemic areas, 65 were Australian human samples (46 negative controls and 19 *Strongyloides* positive) and 7 were dog sera. Two samples from endemic areas were missing. The final numbers of samples tested were 433 by FTS and 454 by QFAT (Figure 1). The country of origin and sample type for the selected endemic samples are shown in Supplementary Table S1.

The numbers of valid tests for the main analyses of concordance and accuracy (endemic areas and Australian controls) were 425 for FTS and 428 for QFAT. The number with valid, matched results for both tests at 10 min was 417. Further details of sample sizes are shown in Figure 1 and Supplementary Figure S1.

### 3.1. Test Validity

At the first 10-min reading, one of the FTS (0.2%, 1/433) and two of the QFAT (0.4%, 2/454) gave invalid results. Tests were successfully repeated for all three samples, providing valid results for 380 FTS tests and 382 QFAT tests from endemic areas (Figure 1). None of the non-endemic samples tested (n = 53 for FTS and n = 72 for QFAT) had invalid results.

### 3.2. Discordance Between Readers

Discordance between the two readers at 10 min with endemic samples was 3.7% for FTS and 1.8% for QFAT (*p* = 0.096). There were no discordant observations reported for non-endemic samples for either FTS or QFAT.

### 3.3. LF Antigen Status Determined by FTS and QFAT

A summary of positive, negative, and indeterminant interpretations for the endemic area samples by sample type and test type at the 10-min reading is provided in Table 1. Overall, the proportion of QFAT results Ag-positive (51.6%; 95% CI 46.4–56.7%) was similar to FTS (45.5%; 95% CI 40.4–50.7%) and had fewer indeterminant results. Table 1 also reports the proportion Ag-positive at the 1 h and 24 h time points. Results by sample type (serum, EDTA plasma, heparin plasma) at the three time points are given in Supplementary Table S2. 

### 3.4. Concordance Between FTS and QFAT at 10 min, 1 h and 24 h Time Points

There was a high level of observed concordance between FTS and QFAT with Kappa values indicating an ‘excellent’ level of agreement, as shown in Table 2. At the 10-min time point, 21 samples were reported as positive by QFAT but negative by FTS, while six were positive by FTS but negative by QFAT. Concordance was found to increase over time with the lowest level of concordance occurring at 10 min (93.5%) and the highest recorded at 24 h (98.8%). Kappa values followed a similar trend with Kappa values increasing from 0.88 (95% CI 0.83–0.92) at 10 min to 0.98 (95% CI 0.95–1.0) by the 24 h time point (Table 2).

Discordance between test results in endemic samples was greatest for EDTA plasma samples using FTS (6.8%) and lowest for serum samples using QFAT (1.0%). These differences were not statistically significant (Supplementary Table S3). However, for EDTA plasma samples at the 10-min time point, the proportion of concordance between FTS and QFAT was still relatively high at 91.6% with an excellent level of agreement (K = 0.83, 95% CI 0.72–0.94); see Supplementary Table S4.

### 3.5. Test Performance Compared to Composite Reference Standard

**Figure 2:**
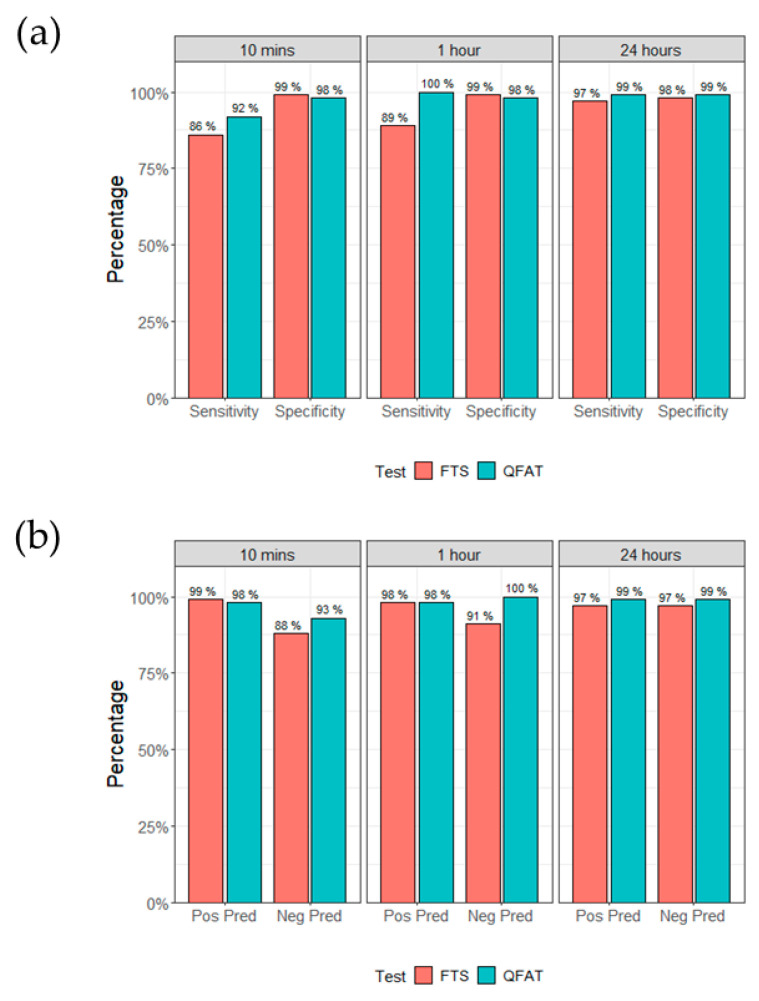
(**a**) Sensitivity and specificity of FTS and QFAT, compared to prior Ag results, at three time points. (**b**) Positive and negative predictive values of FTS and QFAT, compared to prior Ag results, at three time points. FTS: filariasis test strip; QFAT: Q filariasis antigen test; Pos Pred: positive predictive value; Neg Pred: negative predictive value.

At 10 min, when comparing Ag-results from this study with the composite reference standard, the sensitivity, NPV, specificity and PPV of FTS and QFAT were comparable (Table 3). Sensitivity and NPVs for FTS and QFAT increased over time, rising to 99% sensitivity for both FTS and QFAT at 24 h, while specificity and corresponding PPVs remained relatively unchanged, as shown in Figure 2.

### 3.6. Cross-Reactions with Other Helminth Infections

Of the 19 *Strongyloides*-positive human samples, 19 tested with QFAT and 1 also tested with FTS were all Ag-negative. Samples from four dogs infected with *Acanthochilonema reconditum* (n = 1), *Cercopithifilaria bainae* (n = 2), and *Dirofilaria repens* (n = 1) were negative by both tests, while samples from one dog with *D. repens* and one with *Onchocerca lupi* were positive by both tests.

### 3.7. Change in FTS and QFAT Performance over Time

For FTS, four Ag-negatives at 10 min were then reported as Ag-positive at 1 h (0.9%), while a further 18 (4.2%) were reported as Ag-positive at 24 h (Supplementary Table S5a). Most FTS-positives at 10 min remained stable with only one being reported as indeterminant at 1 h but reverting to positive at 24 h (Supplementary Table S5a).

For QFAT, 10 Ag-negatives at 10 min were reported as positive at 1 h (2.2%). Of these, one positive reverted to negative at 24 h. A further two tests were reported to have changed from negative to positive at 24 h for QFAT, while one positive reverted to negative (Supplementary Table S5b).

### 3.8. Line Intensity Scoring of Ag-Positive Tests over Time Stratified by Test Readers

Line intensity score values assigned by the independent test readers were different between FTS and QFAT. Supplementary Figure S2 shows that both observers reported more high-intensity scores using FTS than with QFAT, while the line intensity scores reported for QFAT were mostly low. Particularly for reader 1 of FTS, the proportion of low-intensity scores appeared to increase over time, indicating that test line intensity might fade over 24 h.

### 3.9. Test Acceptability

Two observers independently provided feedback on the tests. Both preferred QFAT over FTS for all aspects except the packaging, which was harder to open quickly for QFAT. Both mentioned that QFAT requires much less sample (10 or 20 µL vs. 75 µL for FTS), was easier to use, and results were clearer to read. QFAT requires an additional buffer step, which was mentioned as a disadvantage.

## 4. Discussion

Our laboratory-based study found a high level of concordance of LF Ag results between the FTS and QFAT. Additionally, it was found that at the initial 10-min reading, QFAT had similar sensitivity and specificity to FTS for detecting *W. bancrofti* Ag, which was determined based on prior known results of LF antigen for samples from the Asia Pacific (Samoa, American Samoa, and Myanmar) [21,22,23,24,25]. Interestingly, it was found that rates of test concordance and sensitivity for both tests increased over 1 h and 24 h. Consistent with our findings in this study, similar levels of concordance between FTS and QFAT have been reported in field laboratory studies conducted in Samoa and India [17,18].

When assessing sensitivity and specificity against prior Ag results (composite reference standard), QFAT was found to have equivalent sensitivity to FTS at the initial 10- minute reading with both tests having similarly high specificity. The concordance between the tests improved at the 1 h and 24 h marks, as did the sensitivity of each test. This trend for sensitivity indicates that this improved concordance rate between the tests was not due to the accumulation of false positives over time. The findings suggest that both tests were robust, and their accuracy might not be compromised if Ag results are interpreted beyond the recommended 10-min reading time. Logistically, this could be useful during LF surveys when the reading time frame needs to be extended during periods of high laboratory workload. Similar changes over time were observed in the field laboratory study [17], but further studies are required to validate this finding, as the field laboratory study had no confirmatory reference standard, and it also showed that QFAT had a significantly higher reported rate of result variation over time relative to FTS, raising concerns about the test’s stability when read at different time points.

Regarding test concordance and sensitivity with different anticoagulant types, we found that plasma in EDTA had a higher sensitivity with QFAT than FTS. False negatives with FTS are a known issue reported with EDTA plasma by field users who routinely use this test in LF surveys. Our results suggest that QFAT does not have this limitation.

Both QFAT and FTS demonstrated cross-reactivity with some dog samples positive for *O. lupi* and *D. repens* but not *C. bainae* and *A. reconditum*. Furthermore, no cross-reactivity was seen for QFAT with *Strongyloides* positive human samples, although we do not know whether these were positive at the time of sampling for antigen as well as antibody to *Strongyloides*. While dog parasites are unlikely to be a significant issue in LF surveys, it is important to consider the potential cross-reactivity with other parasites that could contribute to false positives and interfere with diagnostic accuracy. To further ensure the specificity of these tests, future evaluations should include some additional testing with other human and animal worm parasites, particularly in areas co-endemic with *Loa loa*, where cross-reactivity with FTS has been reported [15].

In this study, we also showed that scoring test-line intensity relative to the control was more informative for FTS than QFAT. FTS frequently had test line scores that were stronger than the control line, while QFAT has a darker control line resulting in usually lighter relative test line densities [17]. These findings are consistent with the field laboratory evaluations in Samoa, in which the test line intensity of FTS was found to be predictive of Mf-status while intensity of line with QFAT was not. QFAT’s darker control improves the readability of the test, reducing the chances of the test being discarded as invalid. While previous studies have suggested that the semi-quantification of test line densities with FTS could be used as a time and cost-effective method for indirectly assessing the levels of Mf-positivity over repeated rounds of MDAs [19,20], this approach does not appear applicable with QFAT.

This study benefited from a controlled laboratory environment and a panel of previously well-characterised samples with Ag status determined by a variety of different diagnostic assays (Og4C3 ELISA as well as ICT and FTS RDTs). The use of a composite reference standard is appropriate for diagnostic studies, as it uses the best available prior information to maximise sample size, as for studies of *Strongyloides* spp. diagnostic tests [26].

We recognize that, as recommended for FTS, most programmes use whole blood for the RDT tests, although many surveys do take anticoagulated blood from finger pricks back to a field laboratory for batch testing. Our study used archived samples and therefore was limited to serum and plasma samples with prior antigen status results. A limitation of this study was that the samples had been stored for long periods of time (several years at −70 °C). Also, tests were conducted on the available samples of serum or plasma, including heparinised or EDTA anticoagulated plasma, which as mentioned previously can impact the diagnostic accuracy of the tests. Our results suggest that QFAT does not have this limitation.

Finally, it was reported that the laboratory users preferred QFAT over FTS in most aspects except for the packaging, which was more difficult to open for QFAT. It was highlighted that QFAT required a significantly smaller sample volume and was easier to set up and read clearly. The need for an additional buffer step for QFAT could be seen as a disadvantage, as this step could be missed accidentally in periods of high laboratory workload, but opinions differ, and some users regard the lack of chase buffer for FTS as a disadvantage. Although the buffer step adds a few extra seconds to the overall time for conducting QFAT, this is balanced by the time needed to wait for the sample to flow to the FTS test strip, so the overall time is equivalent for both tests.

In conclusion, under laboratory conditions, QFAT has comparable diagnostic performance to FTS and is, therefore, a suitable RDT for use in LF elimination programmes in the Asia–Pacific region. Additionally, QFAT has usability advantages over FTS.

## Figures and Tables

**Table 1:** Overall antigen (Ag) status as determined by FTS and QFAT in this study in samples from LF-endemic areas at the 10 min, 1 h and 24 h readings.

Time Point		FTS ^1^ N = 380	QFAT ^2^ N = 382
10 min	N Ag ^3^-positive (%)	173 (45.5%)	197 (51.6%)
	N Ag-negative (%)	199 (52.4%)	184 (48.2%)
	N indeterminant (%)	8 (2.1%)	1 (0.3%)
1 h	N Ag-positive (%)	181 (47.6%)	209 (54.7%)
	N Ag-negative (%)	187 (49.2%)	165 (43.2%)
	N indeterminant (%)	12 (3.2%)	8 (2.1%)
24 h	N Ag-positive (%)	199 (52.4%)	209 (54.7%)
	N Ag-negative (%)	171 (45.0%)	168 (44.0%)
	N indeterminant (%)	10 (2.6%)	5 (1.3%)

^1^ FTS: filariasis test strip; ^2^ QFAT: Q filariasis antigen test; ^3^ Ag: antigen.

**Table 2:** Concordance between FTS and QFAT, excluding indeterminant results, for 10 min, 1 h and 24 h time points.

Time Point	FTS ^1^	QFAT ^2^ Positive	QFAT Negative	N	Concordance (%)	Kappa (95% CI ^3^)
10 min	Positive	167	6	417	93.5	0.87 (0.82–0.92)
	Negative	21	223			
1 h	Positive	180	1	407	94.8	0.90 (0.85–0.94)
	Negative	20	206			
24 h	Positive	196	1	412	98.8	0.98 (0.95–1.0)
	Negative	4	211			

^1^ FTS: filariasis test strip; ^2^ QFAT: Q filariasis antigen test; ^3^ CI: confidence interval.

**Table 3:** Sensitivity, specificity, positive predictive value and negative predictive value of FTS (N = 417) and QFAT (N = 427) at the 10-min reading compared to prior antigen composite reference standard.

	FTS ^1^	QFAT ^2^
Sensitivity % (95% CI ^3^)	86% (80–90%)	92% (88–96%)
Specificity % (95% CI)	99% (97–100%)	98% (95–99%)
PPV ^4^ (95% CI)	0.99 (0.96–1.00)	0.98 (0.95–0.99)
NPV ^5^ (95% CI)	0.88 (0.83–0.92)	0.93 (0.89–0.96)

^1^ FTS: filariasis test strip; ^2^ QFAT: Q filariasis antigen test; ^3^ CI: confidence interval; ^4^ PPV: positive predictive value; ^5^ NPV: negative predictive value.

## Data Availability

The full dataset can be obtained from the corresponding author on reasonable request.

## Supplementary Materials

The following supporting information can be downloaded at https://www.mdpi.com/article/10.3390/tropicalmed10010023/s1. Supplementary Table S1: Profile and number of samples selected for the laboratory comparison of FTS and QFAT for LF antigen detection. The selected samples were collected from LF endemic areas with known LF antigen status (using ICT, FTS or Og4C3 ELISA) determined prior to the current study. Supplementary Table S2: Overall antigen (Ag) status as determined by FTS and QFAT in this study from LF endemic areas at three time points stratified by anticoagulant. Supplementary Table S3. Number and percentage of results with each test that showed discordance between readers at 10 min. Supplementary Table S4A: Concordance of FTS and QFAT at the initial 10-min reading for EDTA plasma samples only. Supplementary Table S4B: Sensitivity and specificity of FTS (n = 95) and QFAT (n = 103) compared with composite reference standard prior Ag result at the initial 10-min reading time point for EDTA plasma samples only. Supplementary Table S5. Samples with reported changes over time (10 min, 1 h and 24 h) in Ag-positivity for (a) FTS and (b) QFAT. Supplementary Figure S1: Flowchart of sample numbers in each comparison. Supplementary Figure S2. Semi-quantitative scoring of test line density for filariasis test strip (FTS) (A) and Q filariasis antigen test (QFAT) (B) of tests grouped by time points (10 min, 1 h and 24 h) and stratified by readers (Observers 1 and 2).
