# Supplementary material for: Laboratory Comparison of Rapid Antigen Diagnostic Tests for Lymphatic Filariasis: STANDARD Q Filariasis Antigen Test (QFAT) Versus Bioline Filariasis Test Strip (FTS)
Source: Trop Med Infect Dis. 2025 Jan 14;10(1):23. doi: 10.3390/tropicalmed10010023 (PMC11768758; doi:10.3390/tropicalmed10010023)

Supplementary File

Supplementary Table S1. Profile and number of samples selected for the laboratory comparison of FTS and QFAT for LF antigen detection. The selected samples were collected from LF endemic areas, with known LF antigen status (using ICT, FTS or Og4C3 ELISA) determined prior to the current study.

**Country**	**American Samoa**			**Samoa**		**Myanmar**	**Total (% of N)**
Year of sample collection	2014	2016	2016	2019	2019	2014	
Sample type	SST Serum	SST Serum	Heparin Plasma	Heparin Plasma	EDTA Plasma	EDTA Plasma	
Prior Ag test used	Og4C3	ICT	ICT	FTS	FTS	ICT	
N with prior antigen results	133	62	62	23	12	92	384
Antigen-positive	8	62	62	23	12	44	211 (54.9%)
Antigen-negative	125	0	0	0	0	48	173 (45.1%)

EDTA: ethylenediaminetetraacetic acid

Supplementary Table S2 Overall antigen (Ag) status as determined by FTS and QFAT in this study from LF endemic areas at three time points, stratified by anticoagulant.

A: 10-minute reading

**Sample Type**	**Serum**		**Heparin Plasma**		**EDTA Plasma**		**Total**	
Test	FTS	QFAT	FTS	QFAT	FTS	QFAT	FTS	QFAT
Ag-positive	54 (27.7 %)	62 (31.8 %)	77 (92.8 %)	79 (94 %)	42 (41.2 %)	56 (54.4 %)	173 (45.5 %)	197 (51.6 %)
Ag-negative	140 (71.8 %)	132 (67.7 %)	6 (7.2 %)	5 (6.0 %)	53 (52 %)	47 (45.6 %)	199 (52.4 %)	184 (48.2 %)
Indeterminant	1 (0.5 %)	1 (0.5 %)	0 (0 %)	0 (0 %)	7 (6.9 %)	0 (0 %)	8 (2.1 %)	1 (0.3 %)
**Total**	**195**	**195**	**83**	**84**	**102**	**103**	**380**	**382**

FTS: filariasis test strip; QFAT: Q Filariasis Antigen Test; EDTA: ethylenediaminetetraacetic acid.

B: One-hour reading.

**Sample Type**	**Serum**		**Heparin Plasma**		**EDTA Plasma**		**Total**	
Test	FTS	QFAT	FTS	QFAT	FTS	QFAT	FTS	QFAT
Ag-positive	54 (27.7 %)	70 (35.9 %)	77 (92.8 %)	83 (98.8 %)	50 (49 %)	56 (54.4 %)	181 (47.6 %)	209 (54.7 %)
Ag-negative	133 (68.2 %)	120 (61.5 %)	5 (6 %)	0 (0 %)	49 (48 %)	45 (43.7 %)	187 (49.2 %)	165 (43.2 %)
Indeterminant	8 (4.1 %)	5 (2.6 %)	1 (1.2 %)	1 (1.2 %)	3 (2.9 %)	2 (1.9 %)	12 (3.2 %)	8 (2.1 %)
**Total**	**195**	**195**	**83**	**84**	**102**	**103**	**380**	**382**

FTS: filariasis test strip; QFAT: Q Filariasis Antigen Test; EDTA: ethylenediaminetetraacetic acid.

C: 24-hour reading.

**Sample Type**	**Serum**		**Heparin Plasma**		**EDTA Plasma**		**Total**	
Test	FTS	QFAT	FTS	QFAT	FTS	QFAT	FTS	QFAT
Ag-positive	67 (34.4 %)	69 (35.4 %)	81 (97.6 %)	84 (100 %)	51 (50 %)	56 (54.4 %)	199 (52.4 %)	209 (54.7 %)
Ag-negative	125 (64.1 %)	122 (62.6 %)	0 (0 %)	0 (0 %)	46 (45.1 %)	46 (44.7 %)	171 (45 %)	168 (44 %)
Indeterminant	3 (1.5 %)	4 (2.1 %)	2 (2.4 %)	0 (0 %)	5 (4.9 %)	1 (1 %)	10 (2.6 %)	5 (1.3 %)
**Total**	**195**	**195**	**83**	**84**	**102**	**103**	**380**	**382**

FTS: filariasis test strip; QFAT: Q Filariasis Antigen Test; EDTA: ethylenediaminetetraacetic acid

Supplementary Table S3. Number and percentage of results with each test that showed discordance between readers at 10 minutes.

	FTS		QFAT		
Sample type	Total tested	Discordant results (%)	Total tested	Discordant results (%)	McNemar's Chi-square p-value
**Endemic samples**					
Total	380	14 (3.7 %)	382	7 (1.8 %)	0.096
Serum	195	6 (3.1 %)	195	2 (1.0 %)	0.221
Heparin plasma	83	1 (1.2 %)	84	1 (1.2 %)	1.0
EDTA plasma	102	7 (6.8 %)	103	4 (3.9 %)	0.371
**Non-endemic samples**					
Total	53	0	72	0	-
Aus neg controls	45	0	46	0	-
Aus *Strongyloides* pos	1	0	19	0	-
Dog serum	7	0	7	0	-

FTS: filariasis test strip; QFAT: Q Filariasis Antigen Test; EDTA: ethylenediaminetetraacetic acid.

Supplementary Table S4A. Concordance of FTS and QFAT at the initial 10-minute reading for EDTA plasma samples only.

**Antigen Status**		**QFAT**		**Total**	**Concordance (%)**	**Kappa 95% CI**
		Positive	Negative			
FTS	Positive	41	1	42		
	Negative	7	46	53		
	Total	48	47	95	91.6	0.83 (0.72-0.94)

Supplementary Table 4B. Sensitivity and specificity of FTS (N= 95) and QFAT (N=103) compared with composite reference standard prior Ag result at the initial 10-minute reading timepoint for EDTA plasma samples only.

**Antigen Test**	**Sensitivity (95% CI)**	**Specificity (95% CI)**
**FTS**	85% (72–94%)	96% (86–99%)
**QFAT**	96% (87–100%)	94% (83–99%)

Supplementary Table S5. Samples with reported changes over time (10 minutes, 1 hour and 24 hours) in Ag-positivity for a) FTS and b) QFAT.

Supp Table 5a: FTS

ID	Country	Sample type	FTS result at 10 minutes	FTS result at 1 hour	FTS result at 24 hours
296	Myanmar	EDTA Plasma	Indeterminate	Indeterminate	Positive
370	Myanmar	EDTA Plasma	Indeterminate	Positive	Indeterminate
271	Samoa	EDTA Plasma	Indeterminate	Positive	Positive
305	Myanmar	EDTA Plasma	Indeterminate	Positive	Positive
327	Myanmar	EDTA Plasma	Indeterminate	Positive	Positive
341	Myanmar	EDTA Plasma	Indeterminate	Positive	Positive
351	Myanmar	EDTA Plasma	Indeterminate	Positive	Positive
328	Myanmar	EDTA Plasma	Negative	Indeterminate	Negative
335	Myanmar	EDTA Plasma	Negative	Indeterminate	Negative
302	Myanmar	EDTA Plasma	Negative	Negative	Indeterminate
356	Myanmar	EDTA Plasma	Negative	Negative	Indeterminate
361	Myanmar	EDTA Plasma	Negative	Negative	Indeterminate
309	Myanmar	EDTA Plasma	Negative	Negative	Positive
354	Myanmar	EDTA Plasma	Negative	Negative	Positive
364	Myanmar	EDTA Plasma	Negative	Positive	Indeterminate
331	Myanmar	EDTA Plasma	Negative	Positive	Positive
86	Am Samoa	HEP Plasma	Negative	Negative	Indeterminate
103	Am Samoa	HEP Plasma	Negative	Negative	Indeterminate
70	Am Samoa	HEP Plasma	Negative	Negative	Positive
88	Am Samoa	HEP Plasma	Negative	Negative	Positive
90	Am Samoa	HEP Plasma	Negative	Negative	Positive
268	Samoa	HEP Plasma	Negative	Positive	Positive

133	Am Samoa	Serum	Negative	Indeterminate	Negative
147	Am Samoa	Serum	Negative	Indeterminate	Negative
151	Am Samoa	Serum	Negative	Indeterminate	Negative
12	Am Samoa	Serum	Negative	Indeterminate	Positive
62	Am Samoa	Serum	Negative	Indeterminate	Positive
128	Am Samoa	Serum	Negative	Indeterminate	Positive
24	Am Samoa	Serum	Negative	Negative	Indeterminate
170	Am Samoa	Serum	Negative	Negative	Indeterminate
7	Am Samoa	Serum	Negative	Negative	Positive
26	Am Samoa	Serum	Negative	Negative	Positive
28	Am Samoa	Serum	Negative	Negative	Positive
43	Am Samoa	Serum	Negative	Negative	Positive
57	Am Samoa	Serum	Negative	Negative	Positive
59	Am Samoa	Serum	Negative	Negative	Positive
129	Am Samoa	Serum	Negative	Negative	Positive
157	Am Samoa	Serum	Negative	Negative	Positive
169	Am Samoa	Serum	Negative	Negative	Positive
130	Am Samoa	Serum	Negative	Positive	Positive
31	Am Samoa	Serum	Positive	Indeterminate	Positive

Supp Table 5b: QFAT

ID	Country	Sample type	QFAT result at 10 minutes	QFAT result at 1 hour	QFAT result at 24 hours
334	Myanmar	EDTA Plasma	Negative	Indeterminate	Indeterminate
371	Myanmar	EDTA Plasma	Negative	Indeterminate	Negative
103	Am Samoa	HEP Plasma	Negative	Indeterminate	Positive
72	Am Samoa	HEP Plasma	Negative	Positive	Positive
74	Am Samoa	HEP Plasma	Negative	Positive	Positive
90	Am Samoa	HEP Plasma	Negative	Positive	Positive
169	Am Samoa	Serum	Negative	Indeterminate	Indeterminate
134	Am Samoa	Serum	Negative	Indeterminate	Negative
145	Am Samoa	Serum	Negative	Indeterminate	Negative
28	Am Samoa	Serum	Negative	Indeterminate	Positive
170	Am Samoa	Serum	Negative	Negative	Indeterminate
141	Am Samoa	Serum	Negative	Positive	Negative
41	Am Samoa	Serum	Negative	Positive	Positive
61	Am Samoa	Serum	Negative	Positive	Positive
62	Am Samoa	Serum	Negative	Positive	Positive
128	Am Samoa	Serum	Negative	Positive	Positive
130	Am Samoa	Serum	Negative	Positive	Positive
133	Am Samoa	Serum	Negative	Positive	Positive
157	Am Samoa	Serum	Positive	Positive	Indeterminate

Supplementary Figure Legends

Supplementary Figure S1. Flow chart of sample numbers in each comparison.

Supplementary Figure S2. Semi-quantitative scoring of test line density for filariasis test strip (FTS) (A) and Q filariasis antigen test (QFAT) (B) of tests grouped by time points (10 minutes, 1 hour and 24 hours) and stratified by readers (Observers 1 and 2).

Supplementary Fig 1: Flow chart of sample numbers in each comparison.

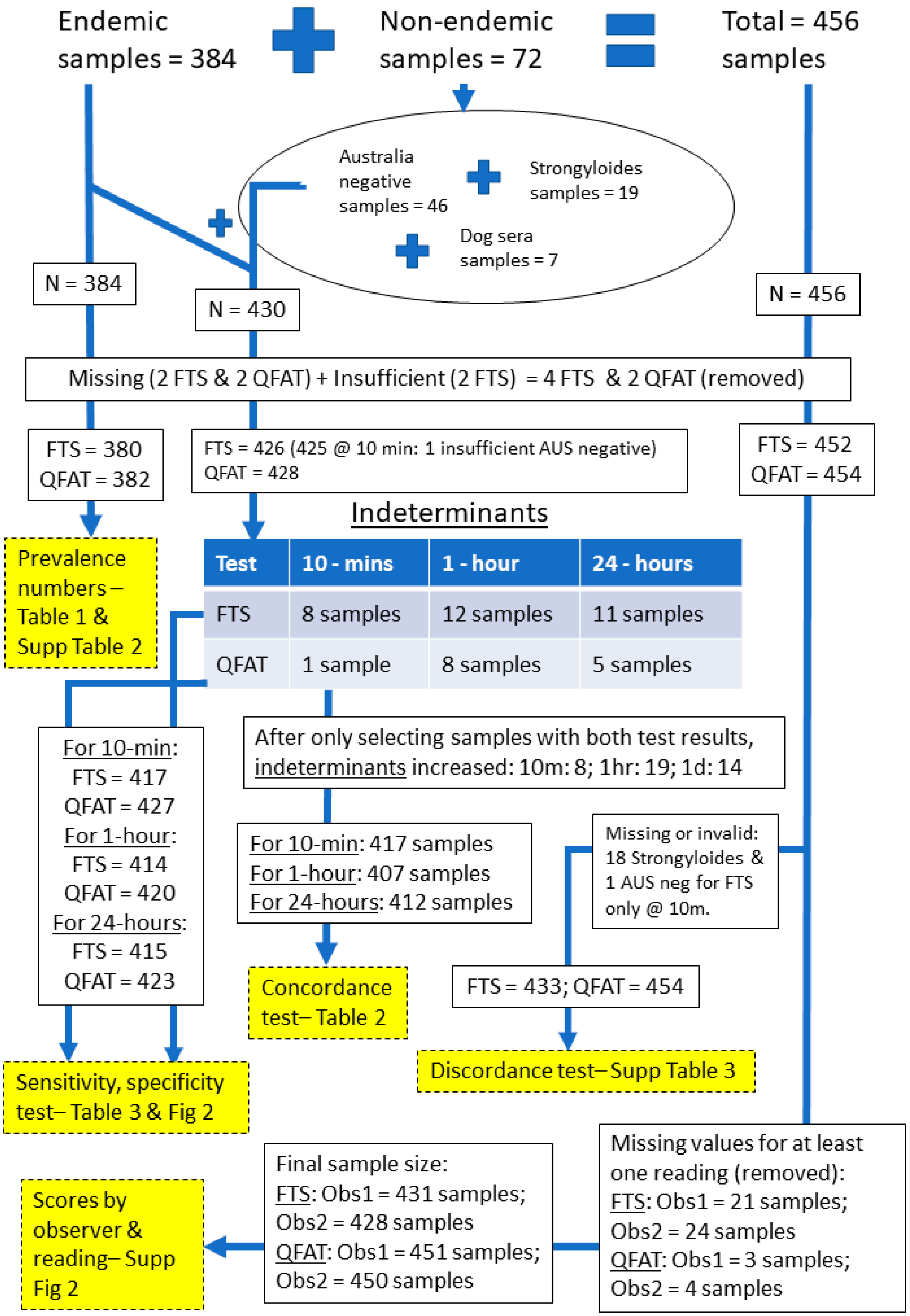

Supplementary Figure S2. Semi-quantitative scoring of test line density for filariasis test strip (FTS) (A) and Q filariasis antigen test (QFAT) (B) of tests grouped by time points (10 minutes, 1 hour and 24 hours) and stratified by readers (Observers 1 and 2).

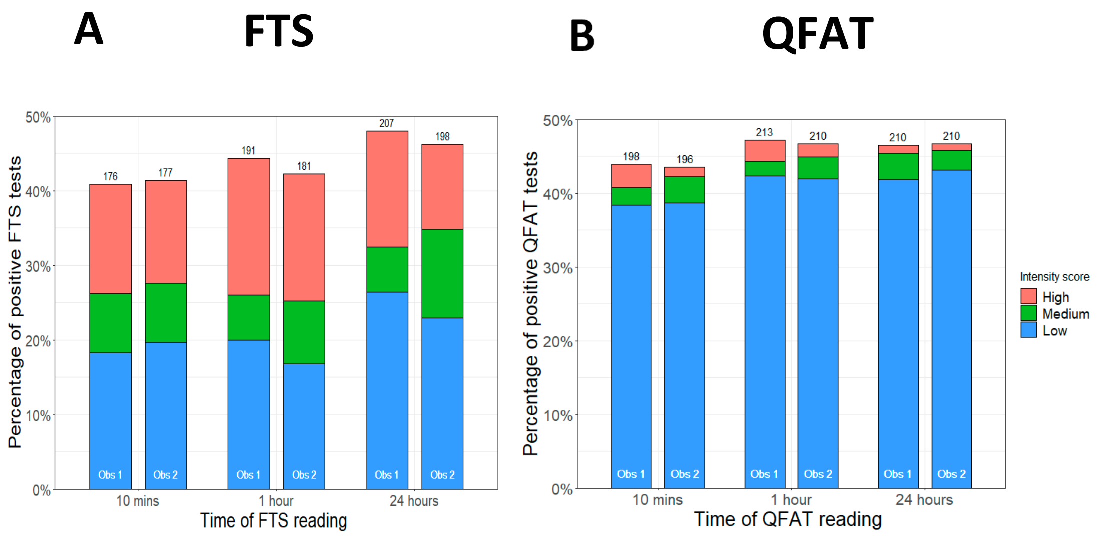

Supplement: Supplementary file 1 [file tropicalmed-10-00023-s001.zip › tropicalmed-3406072-supplementary.pdf]
